# Identification and Characterization of Two Human Monocyte-Derived Dendritic Cell Subpopulations with Different Functions in Dying Cell Clearance and Different Patterns of Cell Death

**DOI:** 10.1371/journal.pone.0162984

**Published:** 2016-09-30

**Authors:** Uriel Trahtemberg, Amir Grau, Adi Tabib, Mizhir Atallah, Alon Krispin, Dror Mevorach

**Affiliations:** The Laboratory for Cellular and Molecular Immunology, Department of Medicine, Hadassah–Hebrew University Medical Center, Jerusalem, Israel; University Medical Center of the Johannes Gutenberg University of Mainz, GERMANY

## Abstract

Human monocyte-derived dendritic cells (mdDCs) are versatile cells that are used widely for research and experimental therapies. Although different culture conditions can affect their characteristics, there are no known subpopulations. Since monocytes differentiate into dendritic cells (DCs) in a variety of tissues and contexts, we asked whether they can give rise to different subpopulations. In this work we set out to characterize two human mdDC subpopulations that we identified and termed small (DC-S) and large (DC-L). Morphologically, DC-L are larger, more granular and have a more complex cell membrane. Phenotypically, DC-L show higher expression of a wide panel of surface molecules and stronger responses to maturation stimuli. Transcriptomic analysis confirmed their separate identities and findings were consistent with the phenotypes observed. Although they show similar apoptotic cell uptake, DC-L have different capabilities for phagocytosis, demonstrate better antigen processing, and have significantly better necrotic cell uptake. These subpopulations also have different patterns of cell death, with DC-L presenting an inflammatory, “dangerous” phenotype while DC-S mostly downregulate their surface markers upon cell death. Apoptotic cells induce an immune-suppressed phenotype, which becomes more pronounced among DC-L, especially after the addition of lipopolysaccharide. We propose that these two subpopulations correspond to inflammatory (DC-L) and steady-state (DC-S) DC classes that have been previously described in mice and humans.

## Introduction

Dendritic cells (DCs) function as the immune system’s sentinels, and are central to its regulation. Upon detection of activating stimuli, DCs undergo a maturation process that encompasses phenotypic and functional changes, which make them the most powerful initiators of adaptive immunity [1]. DCs interact with all cells of the immune system, either directly or through secreted mediators, in both central lymphoid organs and at the immune periphery [2]. DCs can mature in different ways, and their evolution via alternate processes can result in varying effector functions. For example, upon encountering tolerogenic stimuli, the DC response ranges from indifference, to DC apoptosis, to acquisition of a tolerogenic phenotype and function that induces tolerance among other immune cells [3], and that may or may not be accompanied by migration.

DC subpopulations with different characteristics and functions have been identified and shown to perform varying roles. The subpopulations have commonly been defined based on phenotype; however, phenotype is only a surrogate, since it is their specific functions that are of interest for understanding and using DCs. Recent reviews have explored these subpopulations in depth [4–6]. Briefly, murine DCs found in the spleen and lymph nodes have been separated into CD8^+^ and CD8^-^ subtypes, which can be further subdivided. These organs also harbor migratory DCs that come from the periphery. The characteristics of nonlymphoid DCs also vary, with differing characteristics having been described for DCs in various tissues; the skin, gut, and lungs have been studied most frequently. These tissue DCs are commonly initially classified according to their CD11b expression, followed by tissue-specific markers. Distinct from these classical and tissue-resident DCs are plasmacytoid DCs, which specialize in antiviral responses. Finally, while the previously described DCs descend from bone marrow precursors, mdDCs are derived from monocytes.

Our knowledge of human DC subpopulations is less well-developed in comparison with their murine counterparts [6, 7], and the gap between our understanding of mouse and human monocyte derived DCs in particular is significant [8]. In addition, our collective understanding of the extent of correlation between observations of DCs, including mdDCs, *in vivo* and those generated *in vitro*, is far less well understood in humans than in mice [9, 10]. Still, the availability and plasticity of mdDCs make them a prime target for human research [11].

The existence of human mdDC subpopulations has been proposed; however, upon closer examination, it can be shown that the DCs in these studies differed according to the protocols or sera used to produce them [12–16]. In mice, most DCs do not seem to arise from monocytes in the steady state [17]. Indeed, monocytes have been shown to form DCs in inflammation [18–20], but also to reconstitute a portion of intestinal DCs following their ablation [21], and to be incorporated into different tissues as DCs in other studies [18, 22]; thus, the common conception that all murine mdDCs are inflammatory is called into question [5]. In addition, our understanding of human monocyte differentiation into DCs *in vivo* remains a work in progress [23, 24].

The uptake of dying cells is of great relevance for DC function, serving as an important means for DCs to obtain antigens and sample their environment in an everlasting process of peripheral tolerance [25]. Different modes of cell death are associated with signals that influence the DC activation state [26, 27]. In mice, the CD8α^+^ subpopulation specializes in the uptake of dying cells and cross-presentation of their antigens. Human myeloid DCs that are positive for the surface markers BDCA3 (CD141) and CLEC9A are analogous to this subpopulation [28, 29]. These and other works have shown that the context of a cell’s death and its interaction with an ingesting DC can strongly influence the final outcome that the DC itself will effect [26].

We have addressed in this work the death of the mdDCs' themselves, an aspect of DC immunology that has received relatively little attention in the literature. Stimulation of T and B cell for either activation or tolerization is a major role for DCs, and their lifespan is an important regulator of the duration of this stimulus. Common laboratory protocols for T cell expansion use irradiated, mitomycin C-treated, or fixed antigen-presenting cells (APCs), or even completely do away with APCs and use fixed molecular platforms [30]. Some current experiments even use artificial APCs as vaccines [31]. These examples show that injured or even inert APCs and APC-like constructs are functional. Therefore, the study of DC death characteristics is not to be neglected, since it would seem that even dying DCs could have immune effects. Immune cell patterns of death are an integral part of their function, as exemplified by the activation-induced death of T cells. Our group and others have shown that cells committed to die can actively produce immunomodulatory proteins *de novo* [32, 33]. *In vivo*, a DC’s death can have different results depending on its state and location [34]. There are various and conflicting reports on DC death biology, especially on the role of Fas and the bcl-2 family (reviewed in [35, 36]). Nevertheless, it is clear that DC death is a regulated event that is affected by, and also affects, its state and environment.

As described above, monocytes can differentiate into DCs in a variety of tissues and contexts. Moreover, human mdDCs are less well characterized than their murine counterparts. Thus, we asked whether human monocytes can give rise to different subpopulations with different characteristics. In this work we identify and characterize two human mdDC subpopulations, describing differences in their phenotype, morphology and transcriptome, phagocytosis, activation, cell death, uptake of dying cells, and response to dying cell uptake.

## Results

### Forward and side scatter analysis reveals two DC subsets, DC-small and DC-large, which are morphologically different

**Fig 1:**
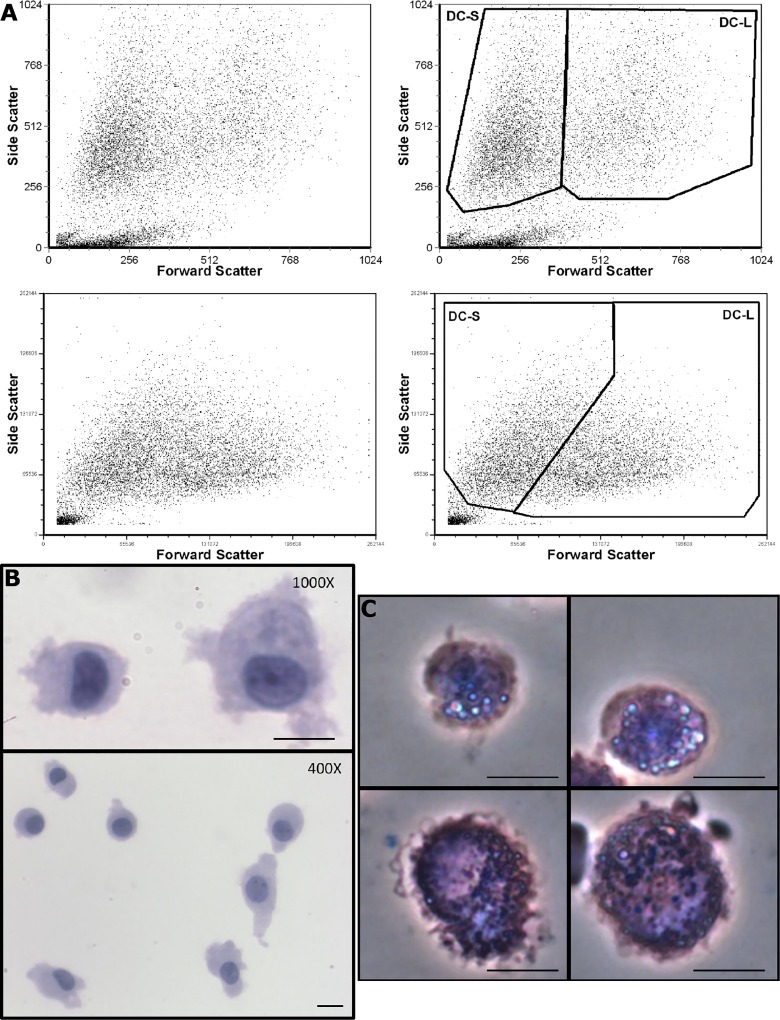
Light scatter and morphology of DC-S and DC-L. A) Forward vs side scatter dot plots of DCs analyzed by flow cytometry. Left panels show the ungated populations, right panels show the gating strategy used. The top panels show iDCs analyzed with FACScan, while the bottom panels show LPS-matured DCs analyzed in an LSR II. Gated populations represent viable cells (see main text). B) iDCs were prepared by cytocentrifugation, fixed with ethanol, and then stained with hematoxylin and eosin. In the top figure, two DCs with significant size differences are seen at high magnification. In the bottom figure, a lower magnification field shows a collection of DCs of different sizes. C) iDCs were sorted as described in Materials and Methods and then imaged live after addition of crystal violet using phase contrast. In the top panels we see two examples of DC-S, while the bottom panels show two examples of DC-L. Bar: 10 μm.

During our work with DCs, we have noticed two clusters of cells on the flow cytometry light scatter plots (Fig 1A), which we have termed “DC-small” (DC-S) and “DC-large” (DC-L). These two populations appeared in all the flow cytometers we used, although they are resolved to varying extents depending on differences in the machines’ light-collecting optics. Among immature DCs (iDCs), DC-S comprise, on average, 54% of the total cells (S1 Fig). After induction of maturation with LPS, the mean percentage of DC-S increases to an average of 61% (S1 Fig). Given that cell death is commonly accompanied by changes in light scatter characteristics, we asked whether these two populations represent different viability states. Overall, less than 5% of DCs are trypan blue-positive on counts, and both DC-S and DC-L are largely viable cells as assayed by Annexin V, propidium iodide (PI), and Sytox Blue (SB) (not shown). These findings were confirmed using DiOC_6_(3), a mitochondrial membrane potential sensitive dye. Thus, cell viability does not account for the DC-S and DC-L differences. Forward scattering is a useful approximation of cell size; accordingly, DCs show significant heterogeneity when imaged, supporting the fact that there are smaller and larger cells (Fig 1B). Upon sorting, morphological differences in DC-S and DC-L are reproduced in the distinct populations (Fig 1C). DC-L are larger, they show greater membrane complexity, and are more granular. Since human monocytes are comprised of two main populations, CD16+ and CD16- [18], we wanted to see whether they were responsible for the development of DC-S and DC-L. To that end, we sorted peripheral blood monocytes into CD14+CD16- and CD14dimCD16+ populations, and differentiated them into DCs using the same protocol. Both monocyte subpopulations gave rise to DC-S and DC-L (not shown).

### DC-S and DC-L express different levels of surface markers and respond differently to maturation stimuli

**Fig 2:**
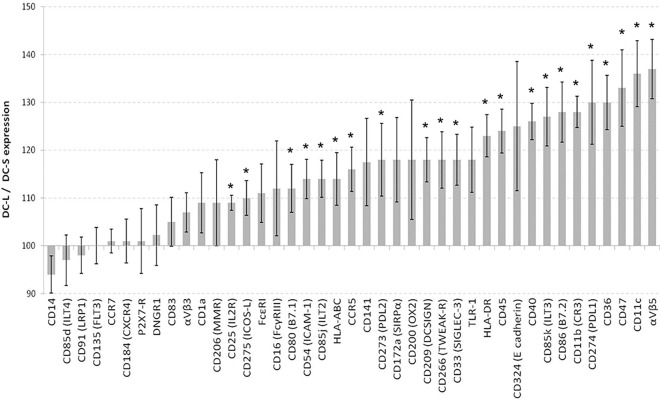
Expression of surface markers on immature DC-L vs DC-S. The relative surface marker expression of DC-L vs DC-S at the immature stage is shown. DC-S median fluorescence intensity (MFI) was normalized to 100; values above and below 100 indicate higher and lower expression, respectively, of DC-L as compared to DC-S. * = p<0.05 for the DC-L / DC-S MFI ratio. n ≥ 3 for all markers. Only SB- or PI-negative cells are shown. Error bars = ±SEM. We also tested CCR2, CD1e, CD121b (IL1R2), CD163, HLA-G, LOX-1 (OLR1), OX40-L (CD252), RAGE, TIM-1, and TSLP-R; however, these surface markers were expressed at very low levels, precluding accurate quantification, or not expressed at all, thus, they are not shown.

We next set out to characterize the expression of surface markers in these two populations using an extensive panel of antibodies. When DCs are immature (culture day 6), the relative expression of surface markers shows a broad spectrum of their relative prevalence, ranging from a DC-L/DC-S ratio of 94-to-135 (Fig 2). Both DC-S and DC-L express CD14 dimly and DCSIGN strongly, indicating that both are fully differentiated DCs. Both DC-S and DC-L express low levels of CCR7, CD83, and CD25, and both upregulate these and other maturation surface markers upon stimulation (see below). This confirms that there are two subpopulations that are initially immature rather than one population of DCs at different maturation stages.

**Fig 3:**
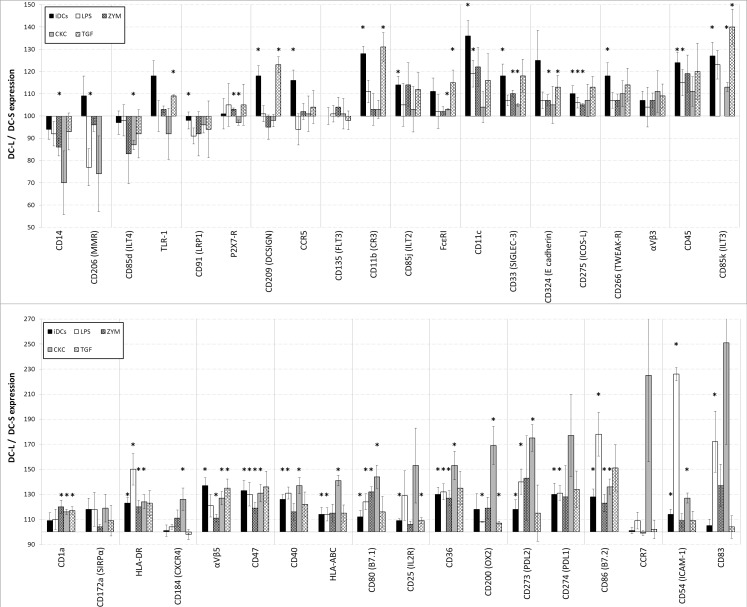
Changes in surface marker expression of DC-L vs DC-S following stimulation. The relative marker expression of DC-L vs DC-S at the immature stage (iDCs), as well as following stimulation with LPS, zymosan, a CKC, or TGF-β is shown. The MFI of DC-S was normalized to 100; values above and below 100 indicate higher and lower expression, respectively, of DC-L as compared to DC-S. * = p<0.05 for the DC-L / DC-S MFI ratio. n ≥ 3 for all markers. Only SB- or PI-negative cells shown. Error bars = ±SEM.

When we tested the DCs following stimulation with a cytokine cocktail (CKC) of PgE_2_, TNF-α and IL-1β; LPS; zymosan; or TGF-β, we noted a striking diversity, and in some cases even a divergence of DC-S and DC-L response patterns (Fig 3). When looking at all the DCs (i.e. before analyzing DC-S and DC-L separately), we see specific responses to LPS and CKC, consistent with the known literature (data not show). Yet when analyzing the subpopulations separately, for both CKC and LPS, as seen in Fig 3, we start to see subset-specific changes. DC-L shows higher expression of stimulatory surface markers CCR7, CXCR4, HLA-ABC, and CD25, or HLA-DR, CD86, and CD54, after CKC or LPS stimulation, respectively. In contrast, stimulation does not alter the DC-L/DC-S ratio of CD135 or α_V_β_3_ integrin expression seen with iDCs. For CCR5, E-cadherin, or CD206, the DC-L/DC-S ratio for iDCs differs greatly from the ratios of all the stimuli used. In other cases, such as DCSIGN or CD11b, the ratio for TGF-β is similar that seen for iDCs (DC-L > DC-S), whereas LPS, zymosan, and CKC move the DC-L/DC-S ratio of these markers towards 100. There are other cases, for example CD14, where the response is markedly different for a single stimulus. While they are immature, both DC-S and DC-L strongly express CD141 (BDCA-3) but low levels of DNGR1. The overall level of expression increases by 50% and 100% for CD141 and DNGR1, respectively, after stimulation with LPS (data not shown), but the DC-L/DC-S ratios remain unchanged at 120 and 95 (Fig 2).

In the S1 File “Raw data examples” we show three examples of individual experiments which are representative of the results shown in Figs 2 and 3.

In summary, upon challenge, DC-S and DC-L show differing responses that are surface marker- and stimulus specific. Importantly, these results are consistent despite the fact that the DCs used here were derived from tens of random human donors whose primary cells underwent up to 8 days of culture during their differentiation and stimulation.

### RNA microarrays reveal a variety of genes that are differentially expressed on DC-S and DC-L

**Fig 4:**
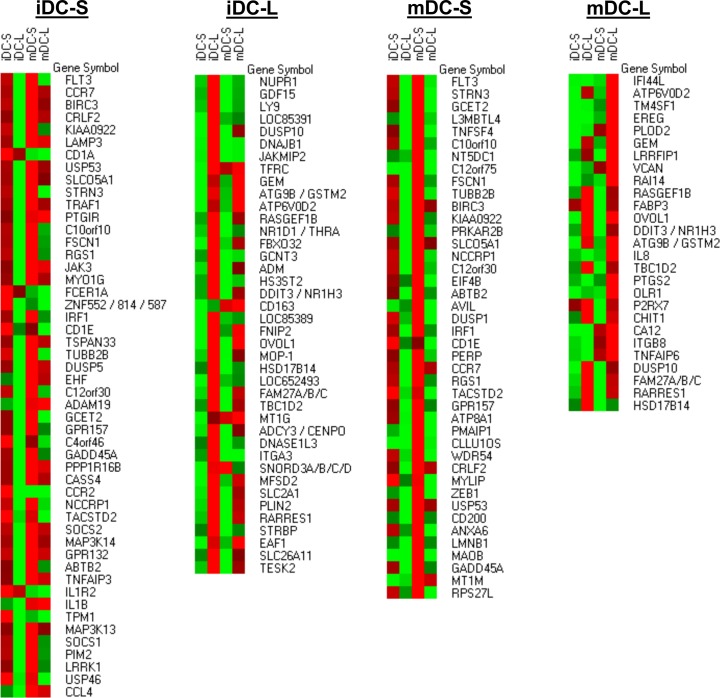
Characterization of differentially expressed transcripts in DC-S and DC-L. iDCs were sorted into DC-S and DC-L and replated for 24 hours with or without LPS, followed by RNA extraction. A pool of 3 experiments was analyzed using Affymetrix microarrays. Four pooled RNA datasets were obtained: DC-S at the immature stage and after LPS stimulation (iDC-S and mDC-S, respectively), and DC-L at the immature stage and after LPS stimulation (iDC-L and mDC-L, respectively). The data was preprocessed using RMA and a cutoff of 4 (log). In order to obtain the list of differentially expressed genes, the expression profiles of DC-S and DC-L were subtracted from each other. The list of genes presented in each category (iDC-S, iDC-L, mDC-S and mDC-L) represents genes that were differentially expressed, defined as a transcript with at least a twofold difference; thus, a gene that is present at similar levels in both subsets would be excluded from the results, even if highly expressed. Due to the cutoff used, fold changes indicate minimal overexpression (the differences can be larger but not smaller). A heatmap representation of the transcripts is shown at absolute levels after RMA and cutoff, in comparison to all the other samples. Red indicates high expression; green, low. Values were row-normalized; shown from top to bottom, from highest to lowest overexpression.

To better understand the nature of the differences between DC-S and DC-L, we analyzed their transcriptional profiles. In Fig 4, we show heatmap representations of the four samples we studied: DC-S and DC-L at the immature stage (iDC-S and iDC-L, respectively) and at the mature stage (mDC-S and mDC-L, respectively). The list of genes of interest was obtained for each of the samples (see legend of Fig 4). Once the list was compiled, we show in Fig 4 the absolute expression level of those genes, in comparison to all the other samples. In the immature DC-S sample (Fig 4), we found several immunologically relevant gene products that are in the bottom third of the DC-L/DC-S phenotypic expression scale among iDCs (shown in Fig 2). This provides an important correlation between the transcriptome and the observed phenotype. Several other immunologically relevant genes are also present in the iDC-S sample. The iDC-L sample also had important immune genes, as well as a large number of genes whose function is not yet completely understood.

When comparing DC-S and DC-L at the LPS-matured stage we see a repetition of genes, showing the stability of their transcriptomes and providing further evidence of their distinct, stable identities. In the mature DC-S sample we find many genes that are not present at the immature stage. In the mature DC-L sample there are a considerable number of immunologically relevant genes as well as an abundance of apoptosis-related genes and genes related to the uptake of dead cells.

### Surface marker expression upon cell death is different for DC-S and DC-L

**Fig 5:**
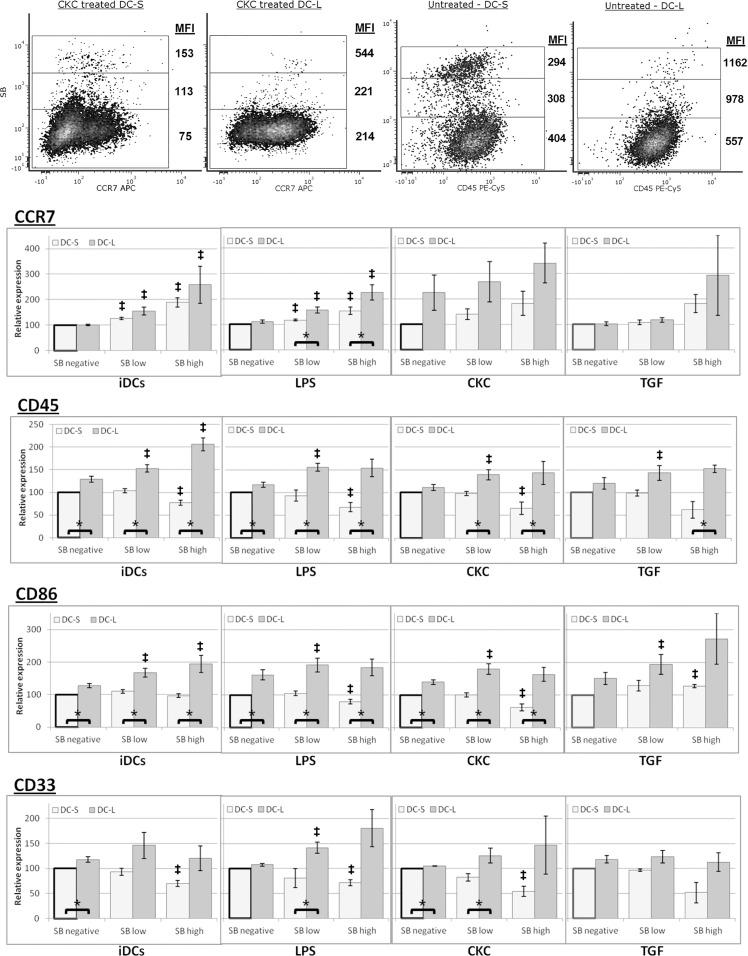
Patterns of surface marker expression changes upon spontaneous DC death. DCs were labeled with fluorescent antibodies for marker expression and co-stained with SB. The cells were gated for DC-S and DC-L, as well as SB negative, low, and high, indicating advancing stages of spontaneous cell death during culture. Top Row: Density plots of representative examples are shown. The MFI of each marker is indicated beside the gates. All gates include at least 50 events. Bottom rows (bar charts): DCs at the immature stage and after stimulation with LPS, CKC, and TGF-β, as indicated, were co-stained with fluorescent antibodies and SB, and gated as described above. Values were normalized so that SB negative DC-S = 100 (bold outline). n≥3 for all markers. * = p<0.05 for the DC-L / DC-S MFI ratio. ‡ = p<0.05 for the DC-L / DC-S MFI ratio change vs SB negative (paired t-test). Error bars = ±SEM.

Since viable DC-S and DC-L differ in surface marker expression, we asked whether there would also be differences upon cell death. To investigate this question, we co-stained all of the samples with PI or SB. It has been shown that PI fluorescence intensity, as well as the intensity of other membrane-excluded, nucleic acid-specific fluorescent dyes, correlates with the advance of cell death [37]. We titrated and tested both SB and PI, including PI+SB double staining, with equivalent results for both dyes. We then classified the cells according to their uptake of SB or PI as negative, low, or high, and measured the MFI of the surface marker of interest for each state (Fig 5). We were surprised to find consistent patterns showing significant differences between DC-S and DC-L phenotypes upon death. As can be seen, when the cells advance in the death process, their level of expression of different markers change. For CCR7, both DC-S and DC-L increase their expression upon advancing cell death. In this case CKC treated cells are shown since they have the highest expression of CCR7 allowing for the clearest visualization. In the case of CD45, in contrast, whereas DC-L still increase the expression levels upon advancing cell death, for DC-S it actually decreases. We identified three general patterns of expression, which are shown in the bar charts in Fig 5: Pattern 1, surface marker expression increases for both DC-S and DC-L as cell death progresses (Fig 5CCR7); Pattern 2, surface marker expression increases for DC-L while it decreases for DC-S as cell death progresses (Fig 5CD45); and Pattern 3, surface marker expression shows a mixed pattern as cell death progresses with behavior dependent on the stimuli used (Fig 5CD86), or surface marker expression does not change monotonically with advancing cell death (Fig 5CD33). Of note, in most cases the mixed pattern (Pattern 3) was similar to Pattern 2. S3 Fig shows these results aggregated for all markers tested in a heatmap representation.

The cells shown represent DCs undergoing spontaneous cell death. It is possible that nonspecific antibody binding could affect the results in dying cells. At the stages of PCD we studied, using a protocol that minimizes nonspecific binding (see Materials and Methods), the antibodies do bind specifically (S2 Fig). When cells enter more advanced stages of cell death, their light scatter properties change, and they exit the analysis gates (Fig 1). PI and SB start entering the cells at an early apoptotic stage, shortly after they become Annexin V positive. They then progressively acquire more PI or SB as they advance in the cell death process. This is the rationale behind the use of PI or SB intensity (in contrast to merely positive vs. negative) as a marker of advancing cell death [37–39]. Of note, experiments performed staining surface markers together with Annexin V and PI or SB showed that there are only very small differences in the surface marker expression when advancing from the Annexin V single positive stage to the PI or SB low stage (data not shown). Therefore, for the sake of simplicity, further experiments were performed using only PI or SB when studying marker expression changes during cell death.

In summary, the expression of surface markers changes upon the DCs’ cell death. This is not a uniform process, with different markers and different stimuli affecting the direction and magnitude of the changes differentially for DC-S and DC-L.

**Fig 6:**
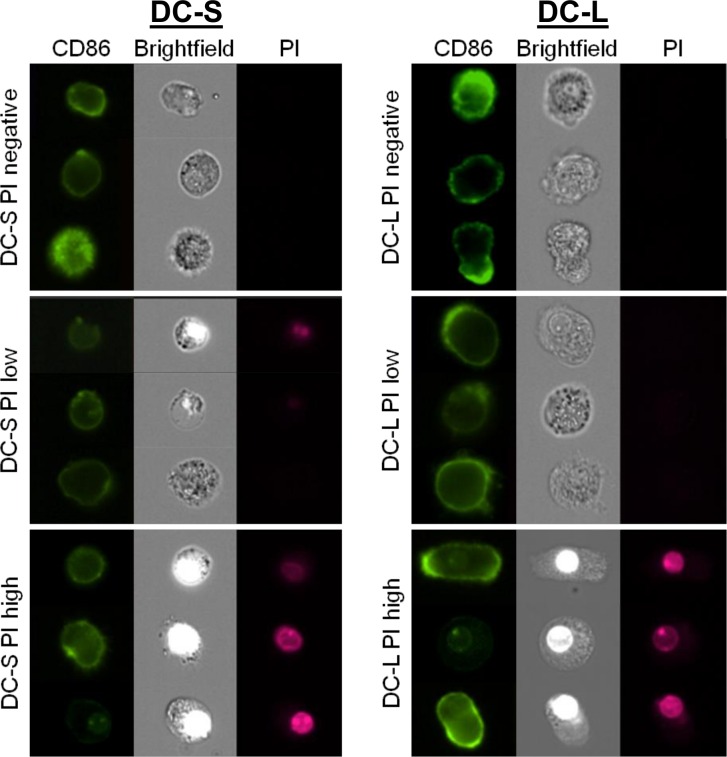
Imaging of live DCs stained with CD86 and PI. iDCs were labeled with CD86, co-stained with PI and imaged using an Amnis Imagestream™ cytometer. The cells were gated into DC-S (left column) and DC-L (right column), as well as PI-negative, low, and high, using an analogous scheme to the one used with other flow cytometers. Three representative examples from every set are shown.

To further assess DC condition at the SB- or PI-low and SB- or PI-high stages, we stained the cells with CD86 and PI, and analyzed them live using an Imagestream™ cytometer (Amnis, EMD Millipore, Seattle, WA, USA). As can be seen in Fig 6, the morphological differences between DC-S and DC-L in size, shape, and intracellular and membrane complexity that were described earlier are confirmed. As the cells advance to the PI-low stage, there are no morphological changes to be observed. This was confirmed by a battery of quantitative morphological measurements provided by the Imagestream analysis software (not shown). This comes to confirm that the PI-low stage indeed corresponds to an early stage of apoptosis. Only at the PI-high stage do we observe morphological changes, such as slight shrinkage of DC-S and loss of membrane and cytoplasmic complexity of DC-L; PI fluoresces strongly in the nucleus, which becomes refractive. Nevertheless, even these "PI-high cells" are whole cells, without blebs and with intact nuclei.

In summary, DC-S and DC-L show differing changes in phenotype upon entering the cell death process. The changing phenotypes are affected by maturation state and stimulus. These changes occur before and at the early phases of acquisition of morphological evidence of cell death.

### DC-S and DC-L have distinct capabilities for phagocytosis, but DC-L is better at antigen processing and uptake of dying cells

**Fig 7:**
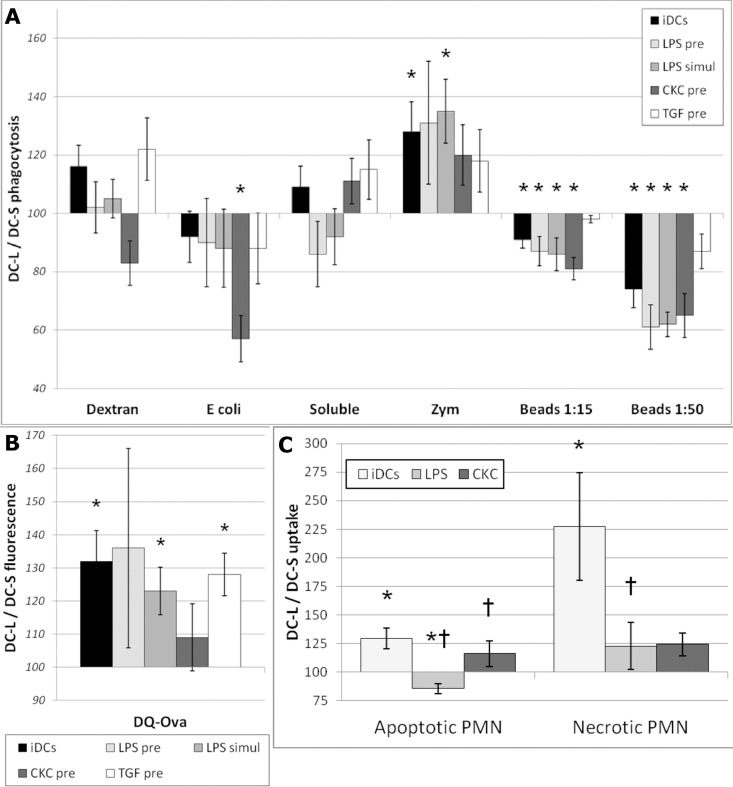
Phagocytosis, antigen-processing, and uptake of dying cells by DC-S vs DC-L. A) Targets were added to iDCs, to DCs previously stimulated for 24 hrs with LPS, CKC, or TGF-β (“pre”), or simultaneously with LPS (“simul”), as indicated. * = p < 0.05 for the DC-L / DC-S MFI ratio. n≥3. Only SB- or PI-negative cells shown. Error bars = ±SEM. DCs were incubated with the indicated fluorescent targets for 8–12 hours and then analyzed by flow cytometry. B) Same as "A" but using DQ-ovalbumin, which is ovalbumin over-conjugated with fluorochrome, and thus self-quenching. After uptake and degradation, the fluorochromes in the resulting peptides are sparser and can fluoresce; therefore, higher fluorescence indicates higher uptake and/or processing of the original protein. C) DCs were incubated with DiD-labeled (fluorescent) apoptotic PMN at a ratio of 1:4 for 8–12 hours. Apoptotic cells were added to iDCs or to DCs previously stimulated for 24 hours with LPS or CKC, as indicated. Samples were then stained with HLA-DR or DCSIGN to specifically identify the DCs, and analyzed by flow cytometry. The MFI of DC-S was normalized to 100; values above and below 100 indicate higher and lower expression, respectively, among DC-L as compared to DC-S. * = p < 0.05 for the DC-L / DC-S MFI ratio. † = p < 0.05 for the DC-L / DC-S MFI ratio change in mature vs immature DCs (paired t-test). n ≥ 3. Only SB- or PI-negative cells shown. Error bars = ±SEM.

To continue exploring the functional differences between DC-S and DC-L, we offered them a variety of fluorescent targets at the immature stage; after stimulation with LPS, CKC, or TGF-β; or simultaneously with LPS. All fluorochromes used are insensitive to endosome acidification. As seen in Fig 7A, DC-S and DC-L do not differ significantly in their capacity for dextran phagocytosis or the pinocytosis of a soluble dye. DC-S show a trend towards better phagocytosis of *E*. *coli*, which becomes significant after maturation with CKC. DC-S also show a significantly better capacity for the phagocytosis of latex beads (except for TGF-β-treated DCs), which becomes more prominent with a higher load of beads. DC-L, in contrast, show a better capacity for uptake of zymosan particles. These results clearly indicate that cell size does not dictate all cellular functions; “bigger” is not always “more”. In Fig 7B we see that DC-L show a stronger signal after being offered DQ-ovalbumin, an assay for antigen uptake and processing. Since both subsets perform pinocytosis similarly (Fig 7A), and since the difference in expression of CD206 (which is a receptor of ovalbumin) is of significantly lower magnitude (Fig 3), this suggests that DC-L is specifically better at antigen processing.

We next gave the DCs fluorescently-labelled apoptotic polymorphonuclear cells (PMN), either at the immature stage or after maturation with LPS or CKC. Fig 7C shows that, even though the differences are not large, DC-L are better at the uptake of apoptotic PMN in the immature stage and after maturation with CKC, while DC-S are better at uptaking apoptotic PMN after maturation with LPS. The DCs were also offered necrotic PMN; surprisingly, uptake by DC-L surpassed uptake of apoptotic cells and was much more efficient than the rates observed for DC-S (Fig 7C).

### DC-L acquires a tolerogenic phenotype after uptake of apoptotic cells

**Fig 8:**
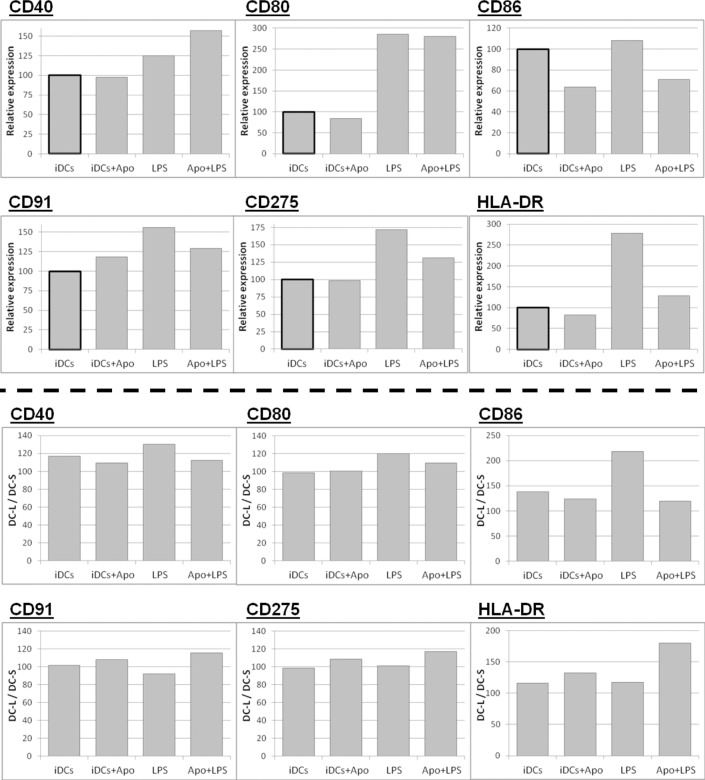
Phenotype after interaction with apoptotic cells. DCs were mixed with apoptotic PBMC at a ratio of 1:4 for 24 hours. LPS was added 6 hours after the apoptotic cells, as indicated. Only SB- or PI-negative cells are shown; representative of 4 experiments. Top panel: The change in the expression of surface markers for all DCs is shown, normalized for iDCs (bold outline). Bottom panel: Same as the top panel, but instead of showing the results for all DCs, the MFI of iDC-S is normalized to 100; values above and below 100 indicate higher and lower expression, respectively, among DC-L as compared to DC-S.

We then set out to assay the differences between DC-S and DC-L following interaction with apoptotic cells. We added apoptotic peripheral blood mononuclear cells (PBMC) to iDCs at a ratio of 4:1 for 24 hours, with or without the addition of LPS 6 hours later. As shown at the top of Fig 8, analysis of data for both sets of DCs reveals strong immunomodulatory effects from the apoptotic cells, with induction of a tolerogenic phenotype at the immature stage and inhibition of the response to LPS (except for CD40). Analysis of DC-S vs DC-L (Fig 8, bottom) shows that after interaction of iDCs with apoptotic cells, the DC-L / DC-S expression ratio was reduced for CD40 and CD86, indicating a decrease of DC-L expression relative to DC-S. Concomitantly, the DC-L / DC-S ratio was increased for CD91, CD275, and, notably, HLA-DR. This pattern was repeated when LPS was added to DCs that had received apoptotic cells, but this time with an even increased magnitude. In order to confirm these results, we repeated these experiments using apoptotic PMN instead of PBMC, with similar results (not shown). Of note, these results show opposite responses of DC-S vs DC-L to LPS as compared to what we found when stimulating them without apoptotic cells (Fig 3).

In summary, apoptotic cells induce an immune-suppressing phenotype among DC-S and DC-L, even after the addition of LPS. Moreover, following interaction with apoptotic cells, DC-L preferentially suppress costimulatory molecules while increasing their relative expression of HLA-DR, a trend that actually increased after addition of LPS to the apoptotic cells.

## Discussion

In this work we present the characterization of two human monocyte-derived DC subpopulations prepared in an autologous system. The subpopulations present distinct morphology, phenotype, phagocytic function, transcriptional profiles, cell death patterns, and responses to apoptotic cells.

Using light scatter we detected DC-S and DC-L, which allowed separating the two populations for flow cytometric analysis using surface markers. After sorting the cells, microscopic results correlated with the flow cytometric light scatter, as did results using the Imagestream platform. However, no single distinguishing surface marker was seen; the differences were in magnitude of expression and response to stimuli. Surface molecules with significant differences in expression included costimulatory molecules such as CD86 and PDL1 as well as HLA-DR; molecules associated with clearance of apoptotic cells such as CD172α, CD11b, αvb5, CD47, and CD36; and after stimulation, maturation markers such as CD54, CD83 and CCR7. These changes could be ascribed to cell size, but given the spectrum of basal expression and changes due to the different stimuli, it is less likely that size is the main determinant of differences between DC-S and DC-L.

Confirmation of these subpopulations' different identities was achieved with transcriptomic analysis. It has been suggested that surface markers can be limited in their ability to differentiate DC lineages, and transcriptomic analysis adds to the characterization of DC subpopulations [40]. Importantly, there were consistent differences between differentially enriched genes and surface markers for DC-S and DC-L, and their identities remained stable after sorting and re-culturing. The salient results from the transcriptomic analysis were, for iDC-S, CD1a and CD1e expression, suggesting a role in the presentation of lipid antigens; in the mature stage, DC-S expression of the combination of TSLP-R, FcER, and OX40L together with lower expression of inflammation-related surface markers, suggests a possible connection to Th2-inducing functions [41]. In addition, DC-S overexpress TNFAIP3, which produces A20, which has been shown to have an important role in maintaining tolerance in the steady state [42]. For DC-L, several genes related to inflammation, such as epiregulin, COX2, P2RX7, OLR1, LRRFIP1, CHIT1, and TNFAIP6 are overexpressed, in keeping with their phenotypic profile.

To support our hypothesis that DC-S and DC-L represent distinct subpopulations, we further examined their responses and functions. In general, DC-L mounted stronger phenotypic responses to inflammatory stimuli. The differing phagocytic capabilities of DC-S and DC-L for various targets provided further reassurance that they are indeed distinct subpopulations. In addition, DC-L better degrade ingested material, which suggests that they may also present it more efficiently. With regard to the clearance of dying cells, DC-L seem to specialize in clearance of necrotic cells, while showing equal to slightly better clearance of apoptotic cells in comparison to DC-S. Apoptotic cells induced an overall state of immunosuppression in the iDC population as a whole (as shown before [43]). It is in this context that a central characteristic of DC-L emerges: after exposure to apoptotic cells, DC-L slightly increase their expression of HLA-DR and reduce CD86 expression; after addition of LPS on top of the apoptotic cells, they strongly magnify these trends beyond the levels seen among the entire iDC population and among DC-S.

The death patterns of DC-L and DC-S are also distinct. The immune significance of DC death has been discussed in the literature [36], but, to our knowledge, differences in cell death have not been used for the characterization of DC subpopulations. It is usually assumed that surface markers are always downregulated upon PCD [44]; however, we found that while the majority of cell markers are downregulated upon DC-S cell death, this is not always the case, and certain surface markers such as CCR7, CD91, and CD80 are actually upregulated. In contrast, DC-L in almost all cases increase expression of surface markers upon cell death. This is an important property that may serve as both a “don’t eat me” and a danger signal (i.e. high expression of HLA-DR and costimulatory molecules) [45]. We have found that there are different patterns of change of the markers upon cell death. This strongly suggests that the mechanism is regulated, supporting the idea that a dying cell is not “inert,” but still undergoes active processes that can be immunologically relevant. DC-S and DC-L show differences not only in scale (as in CCR7), but also diverging responses (as in CD86), further strengthening their separate identities. This is important, since we propose that the death patterns of DC-S and DC-L are different, and that a difference in cell death patterns serves as a novel parameter for the classification of immune cells.

It is usually thought that dying cells cannot or should not be analyzed because of the difficulties and potential artifacts in their handling and analysis, or because of the misconception that by starting the death process they lose their relevance. Yet PCD is an active process that consumes energy and takes time. Our group [33] and others [32] have shown that even cells committed to die carry out active efforts unrelated to their actual self-dismantling. Therefore DCs undergoing PCD may have important effects on their surroundings. DCs carry important information about their environment as well as potentially dangerous antigens, and the context of antigen presentation is a central variable in the initiation of immune responses and in determining the potency of such activation [46–48]. As can be seen in Fig 6, even at a stage of cell death that allows PI internalization, the cells are morphologically unchanged. Only later during the cell death process do morphologic changes of late apoptosis and early necrosis appear, and even then the cells remain intact. Combined with the understanding that even inert particles can cause immune activation (as described in the Introduction), this comes to suggest that dying DCs could also cause immune effects. Therefore, the phenotypic changes seen upon cell death are significant because the dying cells should still be able to induce and affect immune processes. This effect has gone unnoticed in the immunological literature at large when reporting changes of cell surface markers in response to different conditions and stimulations. Thus, an important conclusion of our results, which is applicable beyond the field of DCs, is that regardless of what kind of experiment one is pursuing, it is imperative to add a marker of cell death. Even if there is no interest in studying cell death, failure to exclude dying cells could significantly affect phenotypic readings. One might conclude that a given treatment changes the expression of a certain surface marker, while in reality it is killing the cells.

In light of the specialization of DC subsets, and since monocytes become DCs in a variety of contexts, in this work we asked whether human mdDCs also show a capability for specialization into subpopulations. This is especially relevant since, as described in the introduction, human DCs in general are less well understood than their murine counterparts, and mdDCs are important in the search for DC-based therapeutics. We propose that DC-S and DC-L represent a separation of monocyte-derived DCs into two subpopulations representing two broad types of DCs that were previously described in the literature: inflammatory DCs and steady-state DCs [7, 9]. We suggest that DC-L behave like cells suited to respond to an inflammatory milieu, with their heightened phenotypic response to inflammatory signals and dangerous phenotype upon dying. Their higher capacity for necrotic cell clearance is also significant in such a milieu. Thus, DC-L may represent an inflammatory type of mdDCs [8]. A salient feature of these cells is their “safety mechanism”, evidenced by downregulation of CD86 upon encountering apoptotic cells and further downregulation after the subsequent addition of LPS [49], suggesting a means to avoid autoimmunity.

DC-S express fewer presentation and costimulation molecules than DC-L, and downregulate HLA-DR, HLA-ABC, CD86, and CD200 upon dying; furthermore, after LPS and CKC stimulation they also downregulate CD40 and CD80 upon dying, all of which have important functional implications [50]. DC-S express higher levels of CCR2 and CCR5, indicating a tissue-homing tendency. They also express more Th2-associated molecules such as OX40L, TSLP-R, and FcεRI, and overexpress FLT3, which has been shown to be a key regulator in coupling the inhibitory functions of DCs and Tregs [4]. Therefore, we suggest that DC-S resemble a steady-state, tissue-resident type of DC, with an inclination towards the maintenance of peripheral tolerance. In this respect, it is important to note that they phagocytose apoptotic cells efficiently despite their smaller size, an important characteristic for a steady-state DC.

These two subpopulations, corresponding to two broad DC prototypes, can help us to better understand the role of mdDCs *in vivo*. Moreover, they can be harnessed in therapeutic trials, nudging the desired immune responses either towards inflammation or immunity. In that respect, our work suggests that being able to effectively skew these responses depends not only on designing the adequate context for the antigen-DC interaction, but also on the subpopulation of mdDCs used and their viability state.

## Materials and Methods

### Media & Reagents

Cell culture medium consisted of RPMI 1640 (Invitrogen-Gibco, Carlsbad, CA, USA) supplemented with 1% L-glutamine and 1% penicillin/streptomycin (Biological Industries, Kibbutz Beit-Haemek, Israel). Fluorescent Annexin V was obtained from MBL Inc. (Woburn, MA, USA). Sytox blue (cat S11348), DiD (cat D307), fluorescent dextran (MW 10,000, cat D22910), soluble Alexa Fluor 488 hydrazide (cat A10436), DQ-ovalbumin (cat D82053), fluorescent *E*. *coli* (cat E13231), fluorescent zymosan (cat Z23373), and CFDA-SE (“CFSE”, cat C1157) were obtained from Invitrogen-Molecular Probes. Fluorescent latex beads (cat L5405), DiOC_6_(3) (cat 318426), carbonyl cyanide 3-chlorophenylhydrazone ("CCCP", cat C2759), PgE_2_ (cat P0409), zymosan (cat Z4250), hematoxylin (cat GH5116), eosin (cat H40216), crystal violet (cat C0775), and lipopolysaccharide from *E*. *coli* (cat L6529) were purchased from Sigma Aldrich (St. Louis, MO, USA). Propidium iodide was obtained from both Invitrogen-Molecular Probes and Sigma-Aldrich. IL-4, GMCSF, TNF-α, TGF-β, and IL-1β were purchased from PeproTech Inc. (Rocky Hill, NJ, USA). Primary antibodies were obtained from Dako (Glostrup, Denmark), Becton Dickinson (Franklin Lakes, NJ, USA), BioLegend (San Diego, CA, USA), and AbD Serotec-MorphoSys (Kidlington, UK). CD1e was a kind gift from Henri de la Salle (INSERM U. 725, Strasbourg, France). Secondary antibodies were obtained from Jackson ImmunoResearch (West Grove, PA, USA) and Invitrogen-Molecular Probes. Mouse and goat Ig were from Jackson ImmunoResearch.

### Isolation of PMN and monocytes

The Hadassah-Hebrew University Medical Center Helsinki Committee (which serves as the IRB as well) specifically approved this study, including the use of blood draws and blood bank buffy coat preparations from human participants. The two options for obtaining leukocytes were 1) Buffy coats of healthy donors at the Hadassah-Hebrew University Medical Center Blood Bank (no consent needed, anonymous analysis of donated blood), or 2) Peripheral blood of healthy volunteers (oral consent was obtained, the volunteers were given the opportunity to opt out without penalty, and those who accepted to participate signed a consent form). For the isolation of PMN, RBC were sedimented by adding 6% hetastarch in a 0.9% NaCl solution (Hetasep, Stem Cell Technologies, Vancouver, Canada) and kept at RT for up to 40 min. The leukocyte-rich upper layer of the suspension was then collected and centrifuged on a density gradient using Ficoll (Pharmacia, Uppsala, Sweden). Residual erythrocytes were removed by hypotonic lysis. For the isolation of monocytes, PBMC were prepared using a Ficoll density gradient. Next, positive selection using CD14 magnetic beads was performed according to the manufacturer's instructions (Becton Dickinson). For both PMN and monocytes, purity exceeded 95%, and >95% excluded PI.

### Generation of monocyte-derived dendritic cells

Immature mdDCs were generated from the CD14+ selected fraction of PBMC mentioned above, as described before [43]. Briefly, monocytes were plated in the central wells of 12-well plates at a concentration of 1.25 × 10^6^ / 1.5 mL culture medium, in the presence of 1% autologous plasma, GMCSF (1000 U/mL), and IL-4 (500 U/mL). Every other day, 0.15 mL was removed from the medium and 0.25 mL medium containing plasma, IL-4, and GMCSF was added. iDCs were obtained at day 6. To obtain mature DCs, iDCs at day 6 received fresh media and cytokines together with either 10 ng/mL LPS, 5 μg/mL zymosan, or a CKC consisting of 1 μg/mL PgE_2_, 10 ng/mL TNF-α and 50 ng/mL IL-1β. Alternatively, TGF-β was added at 25 ng/mL.

### Induction and detection of cell death

Viability assays were performed as previously described [39]. Briefly, staining buffer consisted of 140 mM NaCl, 4 mM KCl, 0.75 mM MgCl_2_, and 10 mM HEPES. Annexin V, DiOC_6_(3), PI and SB were titrated to obtain optimal signal to noise [39]. Annexin V and calcium were added 10 minutes before analysis of the cells; calcium was added to reach 1.5 mM. DiOC_6_(3) was added 30 minutes before cell extraction from culture. DiOC_6_(3) was titrated and verified with positive controls using CCCP. To induce apoptosis in PBMCs, they were collected by leukopheresis and frozen. On the day of use they were thawed and exposed to methylprednisolone (Sigma Aldrich), following which they acquired an early apoptotic phenotype (>60% Annexin V+, <5% PI+). To induce apoptosis in PMN, they were incubated at 4 × 10^6^ per mL in 1 mL RPMI in 24-well plates for 14 ± 2 hours, as shown previously [33]. For necrotic PMN, the cells were incubated at 56°C until >80% were trypan blue-positive. For uptake assays, PMN were stained with DiD according to the manufacturer’s instructions, followed by cell death induction as described above.

### Phagocytosis and interaction studies

Phagocytosis targets were added to the DCs for 8–12 hours. The following concentrations were used: soluble Alexa Fluor 488 hydrazide, fluorescent dextran, and DQ-ovalbumin, 1 mg/mL; fluorescent *E*. *coli*, 10 particles per DC; fluorescent zymosan, 3 particles per DC; fluorescent latex beads, 15 or 50 beads per DC. To control for preparation of the samples, control targets were incubated on ice for 30 minutes. DiD-labeled apoptotic or necrotic PMN were added at 4 cells per DC after a washing step. After incubation with the labeled cells for 8–12 hours, samples were stained with either HLA-DR or DCSIGN for specific identification of the DCs and then analyzed using flow cytometry. For interaction studies, apoptotic or necrotic cells were washed and added to the DCs at 4 cells per DC, followed by 24-hour incubation before analysis. When indicated, LPS was added 6 hours after adding the apoptotic cells. To phenotype the DCs after their interaction with dying cells, antibody cocktails were designed so that at least one label would be of a highly expressed DC-specific marker, to exclude free apoptotic cells that entered the scatter gates.

### Cytometry

FACScan™, FACScalibur™ flow cytometers used, and primarily the LSR II™ (Becton Dickinson), and Imagestream™ 100 (Amnis, Seattle, WA, USA). Compensation was performed in software. For microscopy, a Nikon Eclipse E400 microscope equipped with a Micropublisher 3.3 RTV CCD color camera (Q Imaging, Surrey, BC, Canada) was used. Cells were prepared by cytocentrifugation and then fixed in 95% ethanol, followed by a standard hematoxylin and eosin (H&E) staining protocol. Alternatively, the cells were imaged live after adding 10% of a 1 mg/mL solution of crystal violet. We took several measures in order to ascertain the reliability of our results. 1) We performed antibody competition studies, which confirmed that at the stages of cell death studied, antibody binding is specific (S1 Fig). 2) Pulse area vs height doublet discrimination was used to exclude cell pairs that could bias the readings. 3) Isotype controls were extensively used to assess and identify nonspecific binding. Bad samples, identified by abnormally high isotype binding, were excluded. For the remaining samples, we mathematically subtracted contributions of the isotypes (which include autofluorescence) from our calculations. 4) Directly conjugated antibodies were preferentially used. 5) Antibody labelling was performed in the presence of 75 μg/mL of mouse Ig. Alternatively, purified antibodies were used, in which case secondary staining was performed using goat anti-mouse antibodies in the presence of 75 μg/mL goat Ig. 6) The staining buffer consisted of PBS without calcium, supplemented with HEPES and 1% fetal calf serum (all from Biological Industries). 7) As shown in Fig 1A, we were able to gate out terminal apoptotic cells and fragments from the DC clusters.

### Cell sorting

Sorting was performed on a FACSAria I (Becton Dickinson). iDCs were taken at day 6 and stained with CD47, CD11c, and DCSIGN, as well as SB. They were then sorted by creating hierarchical gating that selected DC-S as the cells expressing the lowest levels of all three markers, and DC-L as the cells expressing the highest levels. For monocytes, PBMC were stained with CD14 and CD16, as well as SB. They were then sorted into CD14+CD16- and CD14dimCD16+ populations, and cultured for 6 days as described above for differentiation into DCs.

### RNA & microarrays

Following sorting, the DCs were replated and incubated for 24 hours in the presence of autologous plasma, GMCSF, and IL4. LPS was added at 10 ng/mL when maturation was induced. Then RNA was extracted using Quiagen’s (Hilden, Germany) RNeasy kit according to manufacturer instructions. RNA from 3 donors was pooled and then processed and analyzed at the microarray facility of the Israeli National Strategic Center for Gene Therapy in the Goldyne Savad Institute of Gene Therapy of our institution, using Human Gene 1.0 ST microarrays (Affymetrix, Santa Clara, CA, USA). Preprocessing of the microarray data was done using RMA. Probeset intensities were transformed to s logarithmic scale and a cutoff of 4 was used. Probesets were considered to be differentially expressed if they showed a linear fold change ≥2. Probes lacking refseq identities were excluded, and instances of multiple probes corresponding to the same genes were collated together.

### Data analysis

Software analysis of flow cytometry data was performed using FCS Express (De Novo Software, Toronto, Canada), including software compensation. When studying phenotype upon cell death, relevant isotypes were used and measured as the rest of the surface markers, gated as SB- or PI-high, low or negative (see results). Then, upon summarizing the data, isotype MFI (which includes autofluorescence) was subtracted from marker MFI. Heatmaps were created using “Heatmap Builder”, courtesy of Dr. Euan Ashley (Stanford University School of Medicine). Statistical analysis was performed with Excel (Microsoft, Seattle, WA, USA). The Student’s two-tailed t-test for statistical analysis with a p value of 0.05 for the significance cutoff was used. When applicable, a paired t-test was used, as indicated in the figure legends.

## Supporting Information

S1 Fig FSC vs SSC statistics. S1 Fig shows the proportion of DC-S vs DC-L in graphical form.
(PDF)

S2 Fig Antibody specificity at different stages of cell death. S2 Fig shows the specificity of antibody stains at different stages of cell death.
(PDF)

S3 Fig Summarized patterns of surface marker change upon DC death. S3 Fig shows in heatmap form the complete dataset of the expression patterns of surface markers of DC-S vs DC-L upon (spontaneous) cell death.
(PDF)

S1 File Supplemental Raw Data Examples. This document contains selected examples of the raw data used to compile the statistical results showed in the manuscript.
(PDF)

## Abbreviations

APC: antigen presenting cell
CKC: cytokine cocktail
DCs: dendritic cells
EG: Entrez gene
iDCs: immature dendritic cells.
mdDCs: monocyte-derived dendritic cells
MFI: median fluorescence intensity
PBMC: peripheral blood mononuclear cells
PCD: programmed cell death
PI: propidium iodide
PMN: polymorphonuclears
SB: sytox blue
