# Supplementary material for: Identification and Characterization of Two Human Monocyte-Derived Dendritic Cell Subpopulations with Different Functions in Dying Cell Clearance and Different Patterns of Cell Death
Source: PLoS One. 2016 Sep 30;11(9):e0162984. doi: 10.1371/journal.pone.0162984 (PMC5045195; doi:10.1371/journal.pone.0162984)
Supplement: S1 Fig — shows the proportion of DC-S vs DC-L in graphical form. (PDF) [file pone.0162984.s001.pdf]

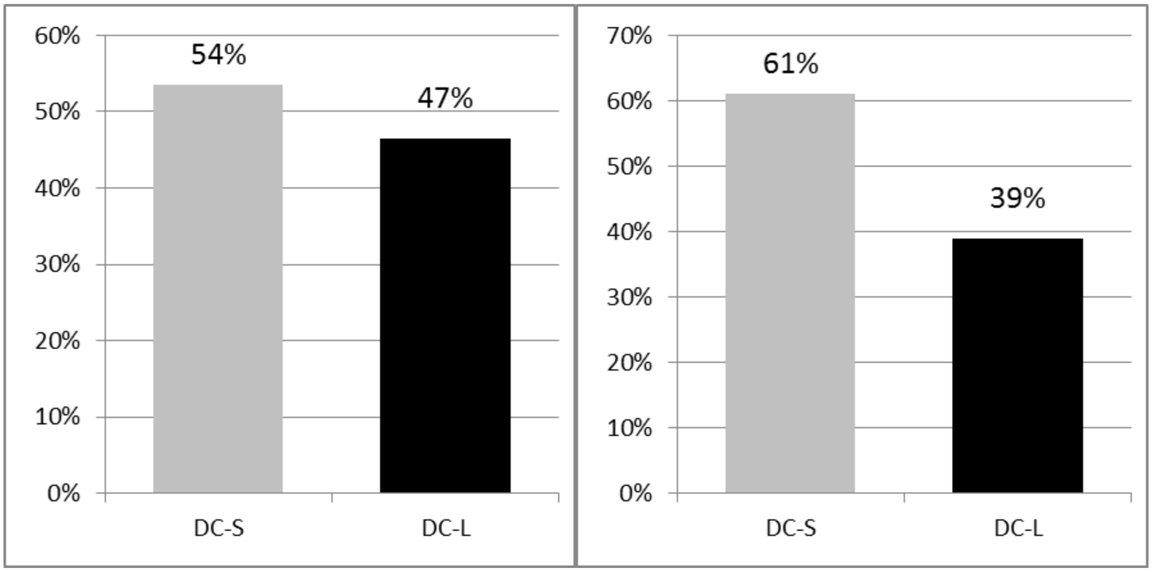

Untreated DCs **LPS-matured DCs**

Supplemental Figure 1 – FSC vs SSC statistics.

iDCs were gates according to the strategy described in the results and Fig 1. The statistics were compiled from 10 matching experiments. For the untreated, iDCs, DC-S are 54% in average, with a range of 46% to 69%, $p=0.018$ for the difference between DC-S and DC-L, $n=10$. After induction of maturation with LPS, the mean percentage of DC-S increases to an average of 61%, with a range of 53% to 71%, $p<0.001$ for the difference between DC-S and DC-L, n-10.
