# Supplementary material for: Identification and Characterization of Two Human Monocyte-Derived Dendritic Cell Subpopulations with Different Functions in Dying Cell Clearance and Different Patterns of Cell Death
Source: PLoS One. 2016 Sep 30;11(9):e0162984. doi: 10.1371/journal.pone.0162984 (PMC5045195; doi:10.1371/journal.pone.0162984)
Supplement: S2 Fig — shows the specificity of antibody stains at different stages of cell death. (PDF) [file pone.0162984.s002.pdf]

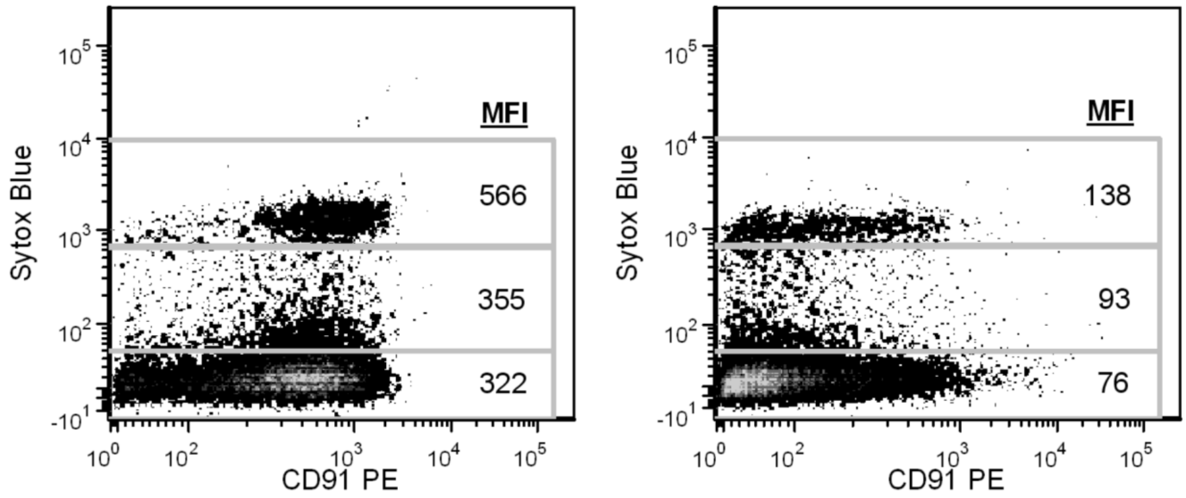

Supplemental Figure 2 – Antibody specificity at all stages of cell death.

iDCs were stained with a phycoerythrin-labeled, anti-CD91 antibody, with (right) or without (left) the presence of unlabeled antibody of the same clone. As can be seen, a reduction in the observed fluorescence of the same magnitude is seen for all stages of cell death (SB high, 76% reduction; SB low, 74% reduction; SB neg, 76% reduction). Similar results were obtained for HLA-DR and CD11c (n=3 for every antibody clone tested).
