# Supplementary material for: Identification and Characterization of Two Human Monocyte-Derived Dendritic Cell Subpopulations with Different Functions in Dying Cell Clearance and Different Patterns of Cell Death
Source: PLoS One. 2016 Sep 30;11(9):e0162984. doi: 10.1371/journal.pone.0162984 (PMC5045195; doi:10.1371/journal.pone.0162984)
Supplement: S1 File — This document contains selected examples of the raw data used to compile the statistical results showed in the manuscript. (PDF) [file pone.0162984.s004.pdf]

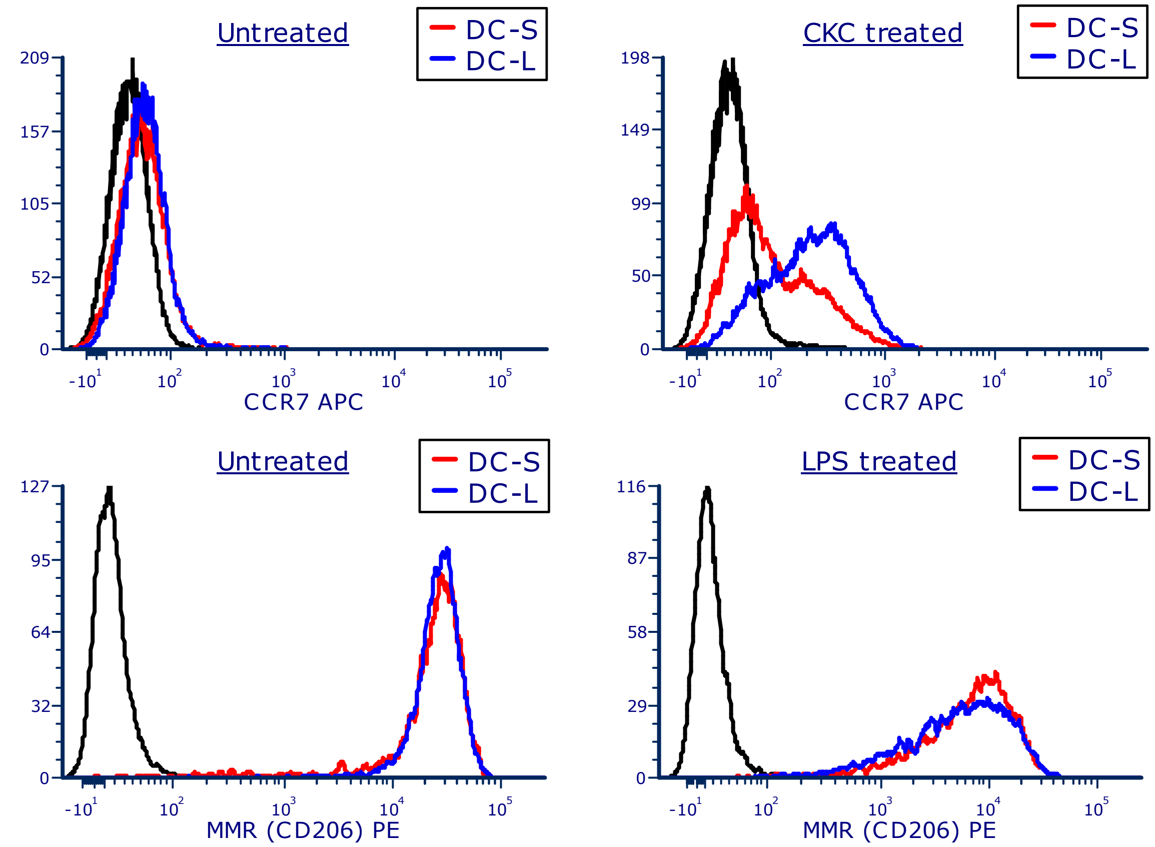

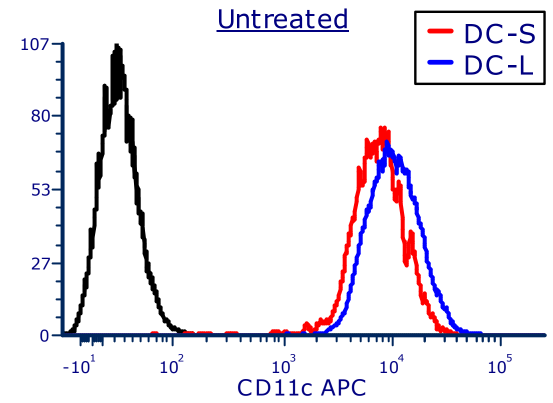

These are representative examples of the data used for the statistics shown in Figures 2 and 3 of the manuscript. Note that the data is shown in a logarithmic scale, where relatively small visual (relative) changes can represent large absolute changes.

1) The top histograms show the expression of CCR7 among DC-S and DC-L, untreated (left) and after treatment with CKC (right). As can be seen, untreated, there are barely a few CCR7 positive cells. After CKC, there is a strong upregulation of CCR7, with an MFI of 75 for DC-S and 214 for DC-L. This corresponds to what is shown in figure 3 - DC-L upregulates CCR7 much more than DC-S. Of note, these are the same files used for the plots shown at the top of figure 5.

2) The middle histograms show the expression of the MMR (CD206) among untreated (left) and LPS treated (right) DC-S and DC-L. Untreated, the MFI of DC-S is 25797 and of DC-L 26466; as shown in figure 2, DC-L slightly overexpresses CD206 vs DC-S among iDCs. After LPS treatement this reverses, with DC-S's MFI at 6996 and DC-L's at 5474; as shown in figure 3, DC-S > DC-L after LPS.

3) CD11c at the bottom of the panels is an example of the largest overexpression of DC-L vs DC-S at baseline, among untreated DCs: the MFI of DC-S is 7117 and of DC-L is 9567.

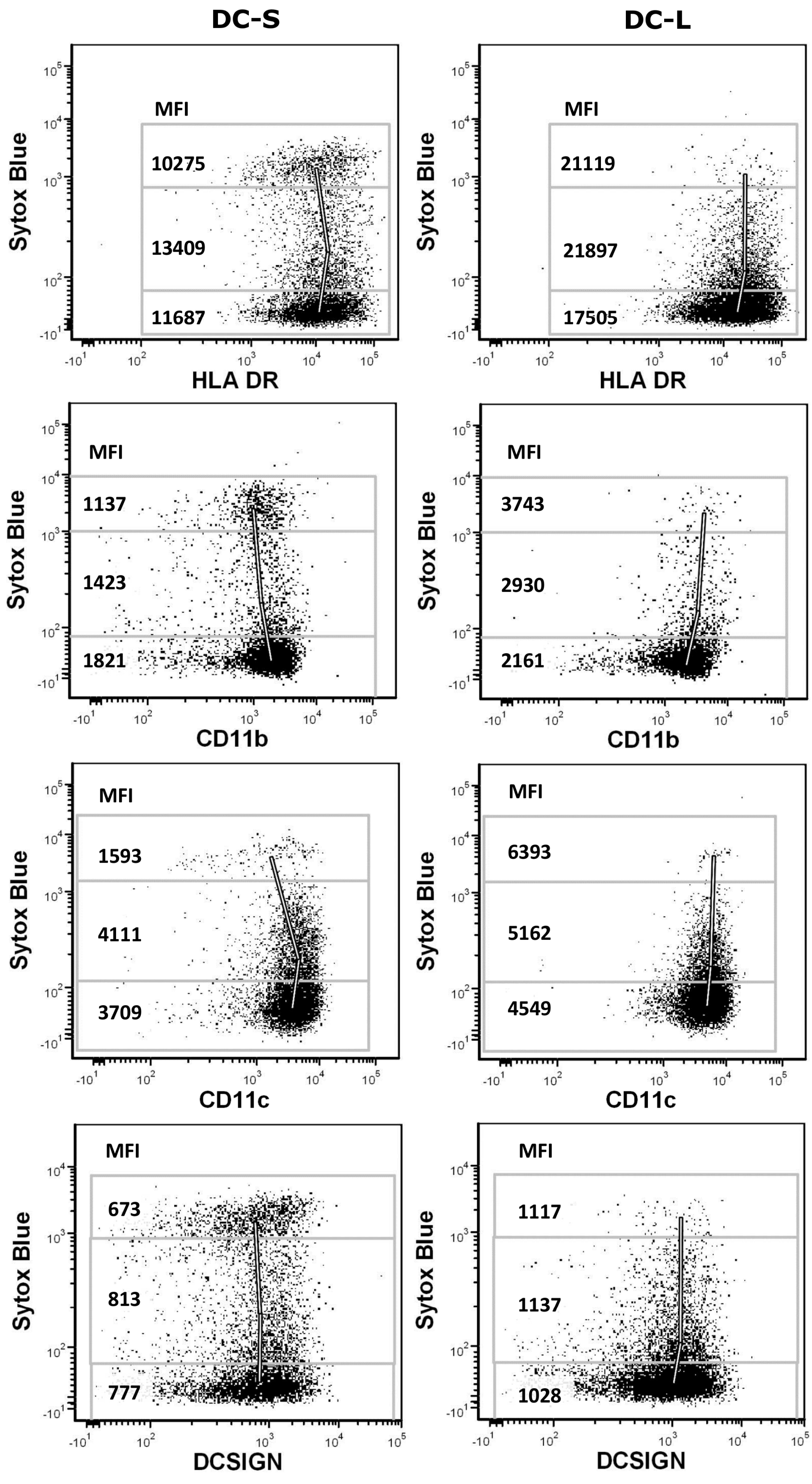

These are representative examples of more cases like those shown at the top of figure 5.

All represent immature DCs undergoing spontaneous cell death. The gates shown represent SB negative, low and high, which are progressive stages of cell death. The MFI of each gate is shown in the figures inside the gates. The lines represent the central trends of the fluorescence as cell death advances (i.e. are the visual equivalent of the MFIs). These all show the most prevalent behavior of surface markers among iDCs: 1) DC-L expresses more of the markers among viable, SB negative cells; 2) upon advancing cell death DC-L increases it's expression while DC-S decreases it.

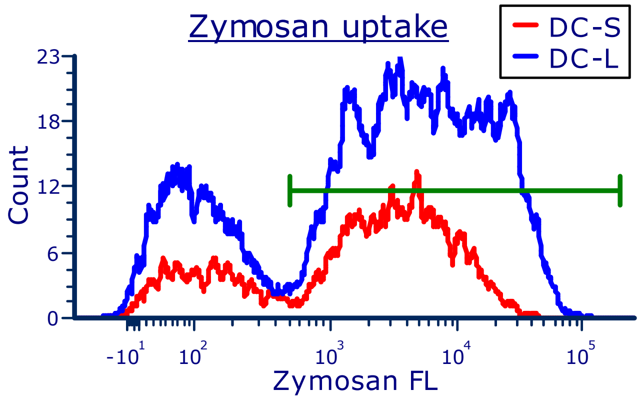

Overlay Gate	Marker	# of Events	% of gated cells	Median	Arithmetic Mean
DC-S	None	3590	100.0	2117.2	3886.3
DC-S	Zym+	2648	73.8	3453.7	5218.8
DC-L	None	9730	100.0	2977.2	7685.3
DC-L	Zym+	7405	76.1	5250.6	10060.4

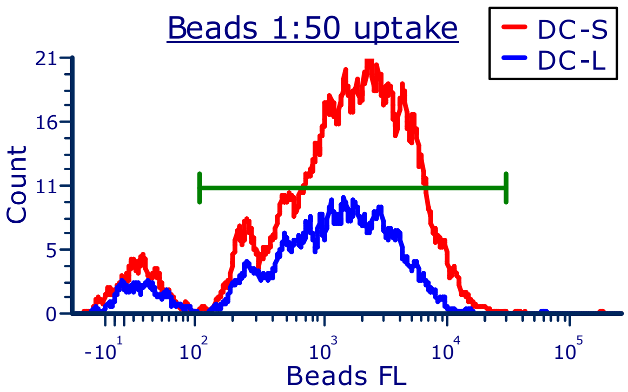

Overlay Gate	Marker	# of Events	% of gated cells	Median	Arithmetic Mean
DC-S	None	5574	100.0	1555.2	2480.8
DC-S	Beads+	5158	92.5	1733.6	2554.3
DC-L	None	2470	100.0	1106.0	1791.2
DC-L	Beads+	2210	89.5	1290.8	1906.1

These are representative examples of uptake of phagocytic targets by SB negative, immature DCs, which are the basis for the statistics shown in figure 7.

Top panel: The percentage of cells uptaking zymosan is roughly similar between DC-S and DC-L, but it is clear that DC-L uptake significantly more particles than DC-S, as shown in the statistics bellow.

Bottom panel: Like for zymosan, the percentage of cells uptaking beads (50 beads offered per DC) is roughly similar between DC-S and DC-L. In contrast to zymosan, though, in this case DC-S uptake significantly more beads than DC-L, as shown in the statistics below. This happens even tough DC-S are smaller than DC-L (as shown in the manuscript, figure 1).
