# Supplementary material for: Identification and Characterization of Two Human Monocyte-Derived Dendritic Cell Subpopulations with Different Functions in Dying Cell Clearance and Different Patterns of Cell Death
Source: PLoS One. 2016 Sep 30;11(9):e0162984. doi: 10.1371/journal.pone.0162984 (PMC5045195; doi:10.1371/journal.pone.0162984)
Supplement: S3 Fig — shows in heatmap form the complete dataset of the expression patterns of surface markers of DC-S vs DC-L upon (spontaneous) cell death. (PDF) [file pone.0162984.s003.pdf]

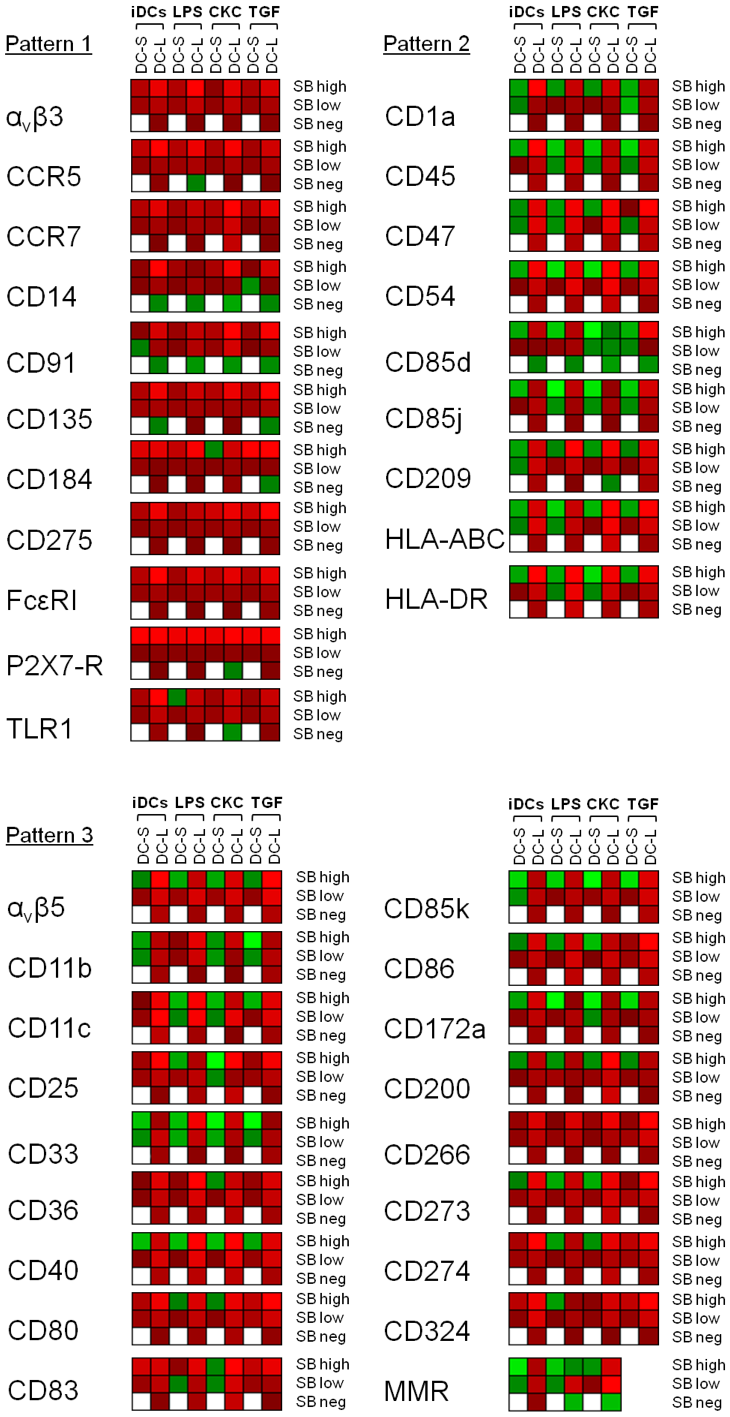

Supplemental Figure 3 – Summarized patterns of surface marker change upon DC death. Heatmap representations of the different patterns of marker expression change upon cell death are shown, as detailed in Fig 5 and 6. Values were normalized so that SB/PI negative DC-S = 100 (white squares). Values above and below 100 are represented in red and green, respectively.
